# A portable bioelectronic sensing system (BESSY) for environmental deployment incorporating differential microbial sensing in miniaturized reactors

**DOI:** 10.1371/journal.pone.0184994

**Published:** 2017-09-15

**Authors:** Alyssa Y. Zhou, Moshe Baruch, Caroline M. Ajo-Franklin, Michel M. Maharbiz

**Affiliations:** 1 Department of Electrical Engineering and Computer Science, University of California Berkeley, Berkeley, California, United States of America; 2 Molecular Foundry, Lawrence Berkeley National Laboratory, Berkeley, California, United States of America; 3 Molecular Biophysics and Integrated Bioimaging Division, Lawrence Berkeley National Laboratory, Berkeley, California, United States of America; 4 Department of Bioengineering, University of California Berkeley, Berkeley, California, United States of America; 5 Chan Zuckerberg Biohub, San Francisco, California, United States of America; The University of Akron, UNITED STATES

## Abstract

Current technologies are lacking in the area of deployable, *in situ* monitoring of complex chemicals in environmental applications. Microorganisms metabolize various chemical compounds and can be engineered to be analyte-specific making them naturally suited for robust chemical sensing. However, current electrochemical microbial biosensors use large and expensive electrochemistry equipment not suitable for on-site, real-time environmental analysis. Here we demonstrate a miniaturized, autonomous bioelectronic sensing system (BESSY) suitable for deployment for instantaneous and continuous sensing applications. We developed a 2x2 cm footprint, low power, two-channel, three-electrode electrochemical potentiostat which wirelessly transmits data for on-site microbial sensing. Furthermore, we designed a new way of fabricating self-contained, submersible, miniaturized reactors (m-reactors) to encapsulate the bacteria, working, and counter electrodes. We have validated the BESSY’s ability to specifically detect a chemical amongst environmental perturbations using differential current measurements. This work paves the way for *in situ* microbial sensing outside of a controlled laboratory environment.

## 1. Introduction

The ability to sense compounds in aquatic environments is indispensable to monitoring water quality, global climate change and chemical threats [1–3]. Traditionally, maritime analysis relied on the manual collection of samples and subsequent analysis on board or after returning to the laboratory [4]. These labor and time intensive processes make it difficult to track trends and offer poor temporal and spatial resolution. To address these challenges, *in situ* chemical sensing techniques have been developed, such as electrical probes, optical spectroscopy, and underwater mass spectrometry [5,6]. Although versatile, sensitive, and capable of *in situ* deployment, technologies such as the underwater mass spectrometer are still relatively power-hungry, large, and require vacuum systems which may disturb the target environment. Even more limiting is the underwater mass spectrometer’s inability to sense non-volatile compounds [2]. Thus, there still exists the need to develop technologies that provide autonomous, deployable, *in situ* monitoring of complex chemicals in aquatic environments.

Since biological systems rapidly detect and report small concentrations of complex chemicals in underwater environments, biosensors are of great interest for *in situ* analysis [1]. The most common type of biosensor utilizes isolated enzymes and antibodies for detection, but these are more suitable for diagnostics, i.e. in-lab detection, as they are only stable in specific conditions, expensive, and susceptible to biofouling [7]. In contrast, microbes are more adept for handling *in situ* environmental sensing by operating across a wide range of pH and temperatures, and having long lifetimes; in addition, they are potentially lower cost [8–10]. Of particular interest for underwater sensing are microbes that produce electrical current, so-called exoelectrogens [11,12]. The electronic signal exoelectrogens produce can be detected with low-power, small footprint devices, unlike optical sensors [13]. A network of these deployable biosensors will also offer better temporal and spatial resolution when monitoring aquatic conditions [4,14,15].

Exoelectrogens can be used to transduce sensing events into electrical signals through two strategies [16]. First, since exoelectrogens need an electron source to produce current, the native microbes can be used to sense a limited number of sugars that serve as electron donors [17]. Second, chemicals can be selectively sensed by engineering the exoelectrogen. In this approach, addition of a small molecule triggers the synthesis of an electron transport protein essential for current production [18]. In this latter method, the exoelectrogen *Shewanella oneidensis* has been engineered to detect the small molecule arsenic [19] and arabinose [20]. Since protein synthesis can be regulated by many small molecules, exoelectrogens should be able to sense various analytes. As proof of concept of our sensing platform, we focus on *S*. *oneidensis* as the sensing element due to its readily available mutants, well defined electron transport pathway, and straightforward growth conditions. Depending on target application, other exoelectrogens, such as *Geobacter* species, may be integrated into the sensing platform.

The sophisticated biological engineering used in these approaches has yet to be matched by the electronics which perform the amperometry and the packaging of the microbes into electrochemical reactors. While there have been recent efforts towards a cost-effective, field-ready potentiostat [5,21], these briefcase-sized devices leave room for further footprint, power, and cost reduction. Additionally, previous prototypes of miniaturized systems still featured large and cumbersome microbe housing, with the system incapable of distinguishing environmental perturbations (pH, temperature, etc.) from presence of a chemical [22–24]. Here we describe a bioelectronic sensing system (BESSY) that leverages the advantages of whole-cell biosensors and state-of-the-art electronics technology to create a deployable, low-power sensor for detection in aquatic environments. We present a novel, miniaturized bioelectrochemical reactor design (m-reactor) which enables engineered bacteria to sample liquid mediums without contaminating the environment. By differentially comparing current production of two strains of electroactive bacteria, a BESSY can specifically detect chemicals amongst other environmental perturbations. This work focuses on a sensing platform for engineered bacteria, which includes the miniaturized potentiostat capable of differential current collection combined with the unique m-reactors. This system demonstrates the potential for whole-cell microbial sensors for *in situ* chemical monitoring in aquatic environments.

## 2. Materials and methods

### Design and construction of miniaturized potentiostat

Our custom potentiostat chip is assembled from components that are all commercially available including (1) a MSP430FG437 low power microcontroller featuring three on chip programmable operational amplifiers, (2) capacitors and resistors for biasing, (3) a 32 kHz crystal for timing, (4) a TS5A23159 analog switch, and (5) input/output ports for connecting to working, counter, and reference electrodes as well as a power supply. The three operational amplifiers each perform a separate task, including (1) buffering the reference electrode potential, (2) driving the working electrode potential +0.2 V with respect to the reference electrode, and (3) amplifying the current signal before recording at the analog to digital converter. The MSP430 microcontroller then outputs this data through serial ports to the RedBear Lab’s WiFi Micro for small footprint wireless communication capabilities. For even lower power consumption, the data could also be stored locally on a SD card. The MSP430 and WiFi Micro chip were programmed using Code Composer Studio and Energia, respectively. Casing for electronics was cast with Polydimethylsiloxane (PDMS, Dow Corning, Sylgard^®^ 184 Silicone Elastomer Kit) in a mold formed with silicone molding rubber (Smooth-On, OOMOO^®^ 300), both mixed according to manufacturers’ instructions.

### Bacterial sensing strains and growth conditions

Four strains of the exoelectrogen, *Shewanella oneidensis*, were used to validate our platform’s sensing capabilities: (1) wild type *S*. *oneidensis* MR-1 [25], (2) a mutant of *S*. *oneidensis* unable to produce current, Δ*mtrB* [26], (3) a mutant of *S*. *oneidensis* unable to reduce fumarate, Δ*fccA* [27], and (4) a GFP-expressing strain of *S*. *oneidensis* containing the p519nGFP plasmid [28]. Cultures were inoculated from frozen glycerol stocks into 250 mL erlenmeyer flasks containing 50 mL 2xYT medium and grown overnight at 30°C with 250 rpm shaking. Fifty μg/mL kanamycin was added to the growth medium for both Δ*fccA* and GFP-expressing *S*. *oneidensis*. After overnight growth, the cells were harvested by centrifugation at 5000g in 4°C for 10 minutes and washed twice with M9 media (Difco^™^ M9 Minimal Salts, 5x). Finally, cells were prepared for injection into the m-reactor by resuspension of the cell pellet in M9 medium until the cell density corresponded to an OD_600nm_ of ~120 (typically 300–400 μL M9 medium added). This high OD is desirable because a large bacterial load in the m-reactors result in higher current signal. This cell suspension was kept on ice until used for reactor construction.

### Design and construction of the m-reactor

To avoid contamination of the m-reactor with non-exoelectrogenic bacteria, it is important for all the abiotic components of the assembly to be sterile and for the construction to occur in a sterile environment. To create the working electrode compartment, conductive carbon felt was shredded to a fine powder and UV sterilized. Next, 250 mg of this carbon felt powder was mixed with 150 μL of the *S*. *oneidensis* cell suspension (OD_600nm_~120) and 1.4 mL of 1% agarose, heated to 55°C, was blended into the carbon felt-bacteria mixture. Then, the carbon felt-bacteria-agarose mixture was compressed into a 2x1x1 cm container, which had been coated with 1% agarose, until the mixture was approximately a half-cm in height. To append the counter electrode compartment, a 3D printed polylactic acid (PLA, a biodegradable thermoplastic) scaffold with a titanium wire wrapped around the topmost part was then inserted until it penetrated the compact mixture. This scaffold improved mechanical stability and ensured the counter electrode wire did not short with the working electrode carbon felt. Lastly, 1% agarose was added to the container until it completely covered the titanium wire (~3 mL). After cooling at room temperature ~15 minutes, or until agarose solidified, the assembly was delicately extracted from the container and a second titanium wire was inserted and secured into the carbon felt-bacteria-agarose mixture to become the working electrode connection. To create a filter, the complete assembly was fully dipped and covered in 0.5% agarose before placing in a desiccator with 8 mL of tetramethyl orthosilicate (TMOS) and left for evaporation for 2 hours. This step deposited thin films of silica onto the assembly for bacterial containment. After removal from the desiccator, the complete m-reactor was placed immediately in M9 medium to prevent drying out. For cases of studies involving portability, we prepared and overnight-air shipped four m-reactors cross-country 4,500 km to collaborators in Washington, DC. The reactors were stored in 50 mL Falcon Tubes with M9 media-soaked wipes padding the reactors. These containers were then packed in Styrofoam containers with frozen gel packs.

#### Visual analysis with confocal fluorescent microscopy

To visualize interactions between the exoelectrogenic bacteria and the conductive carbon fiber, fluorescent microscopic images of the m-reactor were taken on a Zeiss LSM710 confocal microscope with an Axio Observer Z1. Samples of the m-reactor were fixed by submerging in 4% formaldehyde for 20 minutes. Afterwards, samples were washed three times with milliQ H_2_O, and allowed to sit for 5 minutes in milliQ H_2_O between each wash. Samples were stored in M9 medium in a 4°C refrigerator until they were ready to image. Prior to imaging, samples were stained in 14.3 μM DAPI (Thermo Fisher) in M9 medium and 1 μM Cy5 (Thermo Fisher) for 10 minutes at a time. The DAPI stain identifies individual bacteria by their nucleus and the Cy5 provides a nonspecific agarose stain for better carbon felt contrast. Samples were removed from the dye solutions and placed in M9 medium on a glass bottom 6-well plate (Mattek Corporation, P06g-1.5-20-F) and imaged either with a 10x EC Plan-Neofluar objective (0.3 NA) or 100x Plan Apochromat oil immersion objective (1.4 NA). Confocal stacks of the sample were obtained using a 405 nm diode laser to excite DAPI and a 633 nm HeNe laser to excite Cy5, both using a 33.63 μm wide pinhole. Images were falsely colored to enhance contrast for visualizing the sample.

#### Live/Dead analysis

The m-reactor was cut into small samples for live/dead analysis. To create control samples that contained only dead cells, pieces of the assembly were immersed in 70% isopropanol, a disinfectant, for 5 minutes. We used LIVE/DEAD^®^ BacLight^™^ Bacterial Viability Kit (ThermoFisher Scientific, Product number L13152) following the manufacturer’s instructions with slight modifications to accommodate the solid nature of the samples, as follows. One mL of the 5 mL 2X stock solution containing both SYTO 9 dye and propidium iodide was mixed with 1 mL of M9 medium, and then 2 mL of this solution was used to stain the m-reactor. The sample was incubated at room temperature in the dark for 15 minutes and then washed five times with M9 medium before observation. Fluorescent images were acquired on a Zeiss LSM710 confocal microscope with an Axio Observer.Z1 using the 10x EC Plan-Neofluar objective (0.3 NA) with the pinhole set to 33.63 μm. SYTO9, which labels all bacteria, was excited with a 488 nm Argon laser and the emission was detected over the range 493–556 nm (I_SYTO9_). Propidium iodide, which labels only those bacteria with damaged cell membranes, i.e. dead cells, was excited using a 561 nm DPSS laser and its emission was collected between 593–719 nm (I_PI_). All samples were imaged with the same light intensity and detector gain. We calculated the dead:live ratio as R = I_PI_/I_SYTO9_. We used the dead samples as a control for our assembly samples.

#### Colony forming unit (CFU) test

Silica-coated and silica-free m-reactors were prepared and placed in 50 mL Falcon tubes containing 40 mL sterile M9 medium. On the indicated time points (t = 0, 24, 48, 72 h), samples of the medium were taken, diluted with sterile M9 medium, and spread on solid lysogeny broth (LB) medium containing 1.5% agar. Plates were incubated overnight at 30°C and resulting colonies were counted.

#### Scanning electron microscopy analysis

Thin slices of the m-reactor were cut and placed in Tris buffer (pH 7.5). Glutaraldehyde to a final concentration of 2.5% (v/v) was added to the slices and incubated for 20 min to fix the samples. Serial dilutions of ethanol (10, 25, 75, 90, 100% ethanol) were applied at 15 minute increments to dehydrate the sample. The slices were placed in closed petri dishes and left to dry overnight in a fume hood. Specimens were sputtered with gold to an approximate thickness of 10 nm prior to visualization. The field emission scanning electron microscope used was the FESEM Ultra 55 set to an EHT (extra high tension) voltage level of 3–5 kV, under vacuum (5.0x10-5 mbar), and working distance of 4–7 mm.

### Electrochemical setup and environmental conditions

Experiments were conducted in 1 L beakers full of M9 medium. Three-electrode potentiostat measurements were taken by connecting the working electrode and counter electrode titanium wires of two m-reactors to a single custom PCB device capable of two-channel monitoring. We set the potential of the working electrode of each active reactor at +0.2 V with reference to a 3M Ag/AgCl reference electrode. As proof of system functionality, 40mM lactate was added to a wild type/*ΔmtrB* BESSY. To exhibit the fumarate specific sensitivity of the BESSY, 10 mM lactate and 1 mM fumarate were added in the wild type/*ΔfccA* BESSY. Extreme temperature fluctuations were introduced by immersing the beakers in ice baths for 8 hours, and removing to room temperature at benchtop.

### Data collection and processing

The custom potentiostat was programmed to collect current readings every 15 seconds. The data was transmitted through RedBear Labs Wifi Micro board to a Google Documents spreadsheet using Temboo, Inc’s commercially available WiFi Choreos. Every 10 minutes, the device switched between the two potentiostat channels such that only one was active at a time, to prevent any crosstalk between the reactors. Every time a channel was switched on, there was a period of exponential decay when the cells expelled a buildup of charge through the capacitive-like membrane, which accumulated during the period of inactivity. Therefore, the raw data was fit to an exponential decay curve for each 10 minute set of data. From these fitted curves, the cells steady state current output was extrapolated. The data was then passed through a five-point moving average to reduce noise. This data processing, in addition to separating the two channels, allowed us to compare the steady state current fluctuations between the two strains of *S*. *oneidensis* in response to different environmental stimuli.

## 3. Results and discussion

### Custom potentiostat reduces volume typically required for electrochemical analysis

**Fig 1:**
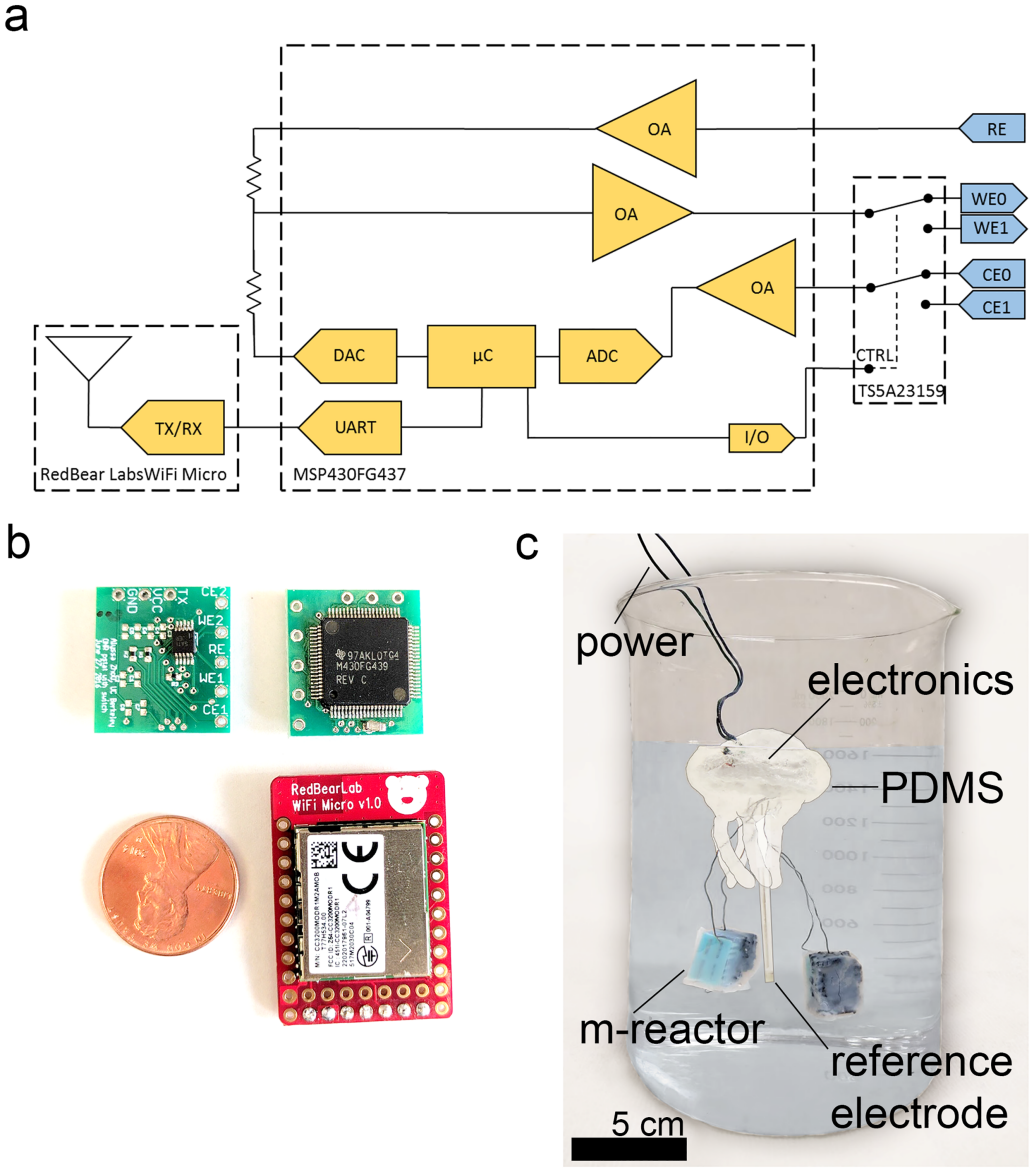
A miniaturized BESSY for deployable electrochemical monitoring. (a) Circuit diagram of a commercial, off the shelf (COTS) potentiostat featuring three on chip operational amplifiers on a MSP430FG437 microcontroller sending serial data to Redbear Labs Wifi Micro board for wireless data transmission. Only circuitry for one three-electrode channel is on board, but switching results in multiple channel potentiostat function. (b) Final potentiostat PCB (green) footprint is 2x2 cm and is connected to WiFi micro board (red); penny is shown for size comparison. (c) Contrast corrected photograph of the BESSY for differential sensing and deployment in aqueous environments.

Realizing a deployable sensor capable of *in situ* detection requires miniaturization in both the abiotic and biotic components. In addition, the abiotic sensing structure must be capable of independently sampling two channels for differential current analysis without crosstalk, and transmitting the sensor readings to a user. To this end, we miniaturized the traditionally bulky and heavy electrochemical analysis equipment to a 2x2 cm printed circuit board (PCB). This board serves as a two-channel, three-electrode potentiostat. The potentiostat is configured such that only one channel is operational at a time and alternates between both channels (Fig 1a), thus avoiding crosstalk between the two channels induced by current collection on the same reference electrode. Although the frequency of switching can be adjusted, for this study the system measures each channel for ten minutes at a time. This time is approximately how long it takes for the measured current to settle to steady state after each switch (measurements are taken at fifteen second intervals). This switching of channels allows for two independent electrochemical measurements to be taken with the machinery of only one channel, reducing power and footprint. Moreover, this switching allows a single device to sense the target analyte in the environment in the presence of other variables by taking differential measurements between a responsive and a null strain. The custom PCB is united with a RedBear Labs WiFi Micro module for wireless data transfer, both components shown in Fig 1b. Together, the platform consumes 30mW when sampling and transmitting information once every 15 seconds, with the majority of the power going to WiFi communications. When compared against a commercial potentiostat (BioLogic VSP-300), we found that the COTS potentiostat produced values within 10% of the commercial unit over the range of 10μA to 1.5mA (S1 Fig). This reduction in accuracy and range is traded off with a greater than 100x decrease in price ($60 as opposed to $6000), as well as an over 4000x decrease in volume (7 cm^3^ as opposed to 30,000 cm^3^). Due to the use of reliable, commercial components, we see 100% yield in production of these potentiostats. The electronics are packaged and sealed in a buoyant, biocompatible, waterproof PDMS casing which protects the electronics and allows the system to sample environmental conditions of an enclosed body of water (Fig 1c).

### M-reactor increases portability

**Fig 2:**
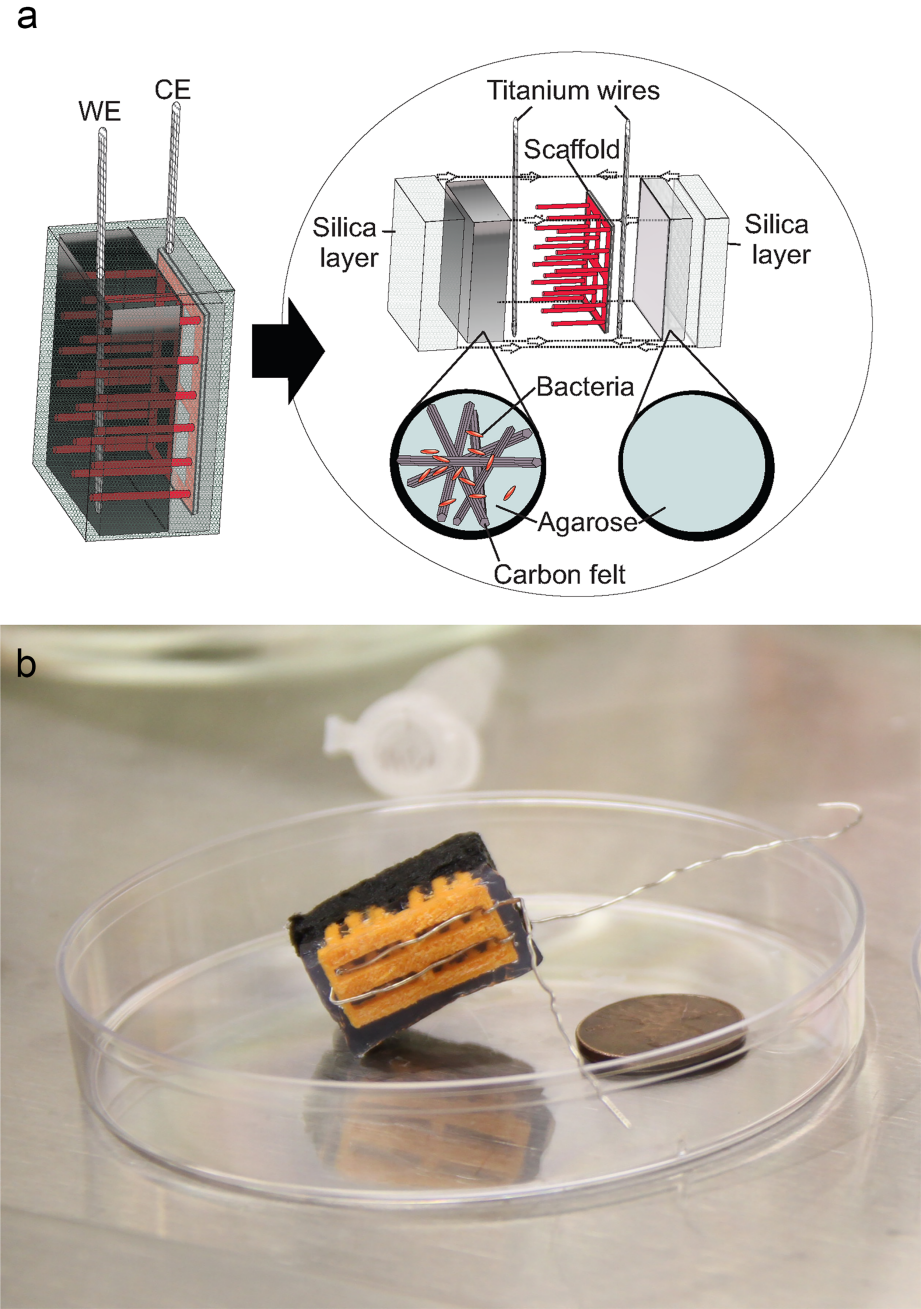
M-reactor for microbial integration. (a) Conceptual schematic of electrode detailing how the scaffold stabilizes the bacteria containing, carbon felt, working electrode (WE) compartment (black) with the nonconductive, agarose, counter electrode (CE) compartment (gray) then coated with a silica layer. Titanium electrodes are partially embedded in agarose for contact with the carbon felt and partially emerge from the reactor complex for attachment to the electronics. (b) Realization of m-reactor with working electrode compartment (black) contained in a 1% agarose mixture stabilized with a 3D printed PLA scaffold (orange); penny is shown for size comparison.

The m-reactor is designed to collect current from a living bacterial strain that is sampling the environment. To achieve this, (1) the bacteria must survive the fabrication process and be housed in an environment conducive to cell viability, (2) the bacteria must be within electron transfer distance of a charge collector, and (3) the bacteria must be surrounded by a size-selective filter to prevent their release, yet permit diffusive transport of small molecules. Thus, we designed an assembly in which a 1% agarose gel contains carbon felt fibers and the bacteria, which is electrically connected to a titanium wire, creating a working electrode. This working electrode is separated by ~5mm from a titanium wire counter electrode which is also embedded in agarose. An evaporated silica layer encases both electrodes (Fig 2). The fabrication process described in Section 2 allows for (1) custom geometries through molding, (2) room-temperature, non-desiccated assembly, and (3) a size-selective filter using tetramethyl orthosilicate (TMOS) silica deposition, thus obviating the need for chemical or physical sealing of reactor chambers.

**Fig 3:**
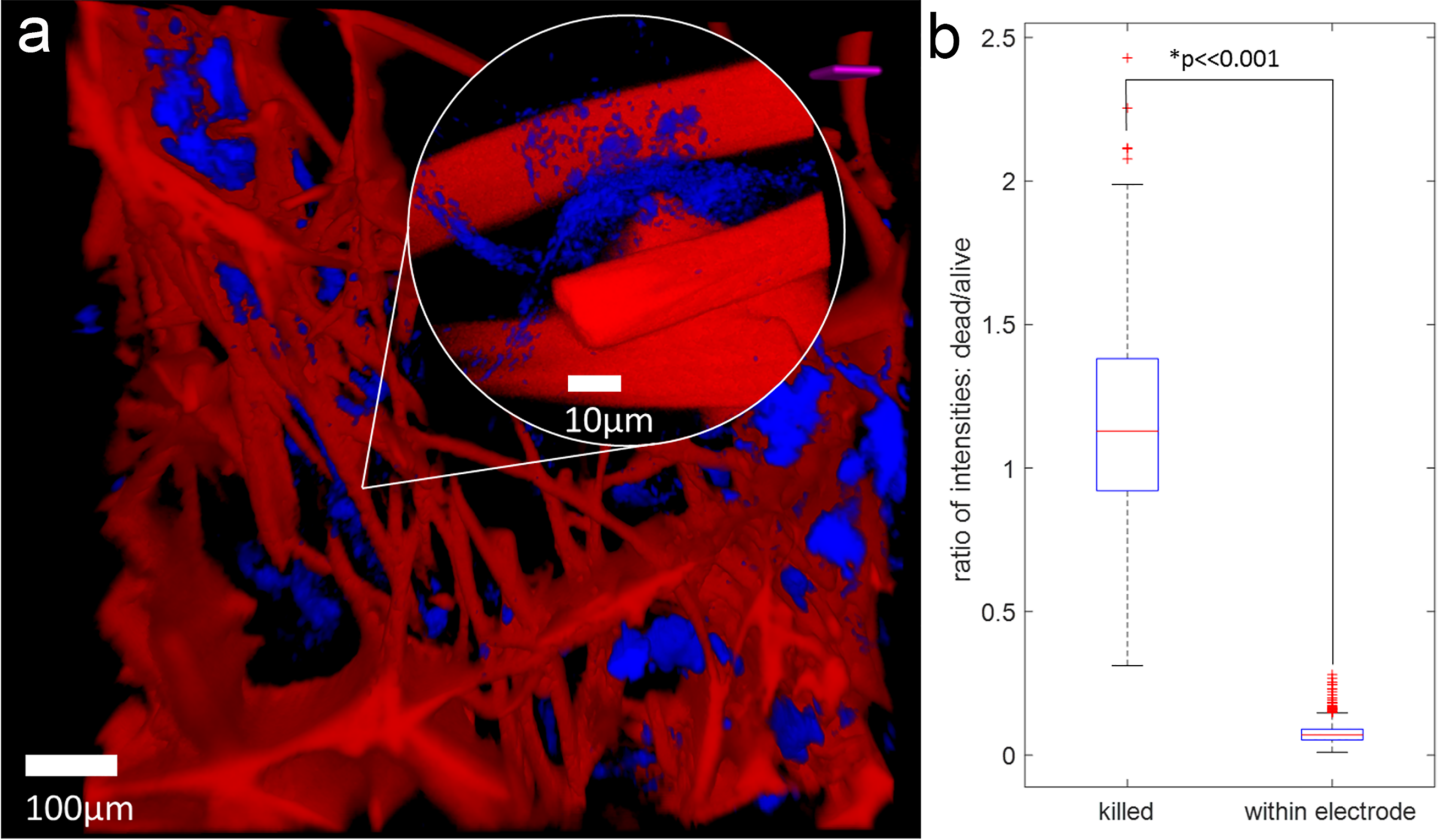
Microbial viability following m-reactor construction. (a) Fluorescent confocal microscopy image of electrodes to visualize internal interaction of bacteria (blue) with carbon felt (red). Inset shows a closeup of bacteria intertwining the conductive carbon felt, illustrating <10 μm distance between the exoelectrogens and felt. Inset is a representative image of a closeup of the bacteria, and is not a blowup of an exact region on the larger image. (b) Distribution of the ratio of intensities for control dead cells (N = 173) and samples of our reactor (N = 685). The p<<0.001 chance of similarity indicates that bacteria in the m-reactor are not dead.

In order to visualize how the bacteria are distributed among the conductive fibers in our m-reactor, we stained the bacteria with DAPI and Cy5 and took fluorescent confocal images of our constructed electrodes. These images (Fig 3a) suggest that the bacteria are randomly aggregated in tight clusters around the conductive fibers, which allows them to deliver current to the carbon fibers. As seen by their positioning around the carbon felt, a significant number of *S*. *oneidensis* are within 10 μm, which should allow for electron transfer by three proposed mechanisms: direct electrode contact, soluble flavin mediators, and contact through conductive nanowires [29–32].

To determine the survival rate of the bacterial population through this construction process, we performed live/dead assays (see Materials and methods) on the bacteria in the electrode. The bacteria in isopropanol-sterilized control samples had an average fluorescent intensity ratio of 1.16 (n = 173). This near unity dead:live fluorescent ratio is consistent with these cells being dead since the SYTO 9 stain labels all bacteria, regardless of membrane quality whereas propidium iodide only penetrates bacteria with damaged membranes, i.e. dead cells. In contrast, the fluorescence intensity ratio in the samples from the m-reactor averaged 0.07 and had no overlap in distribution with the isopropanol control (Fig 3b), indicating that the sampled cells were not significantly disrupted (n = 685, p<0.001), and had intact membranes. This confirms the described m-reactor construction process enables encapsulation of viable microbes.

**Fig 4:**
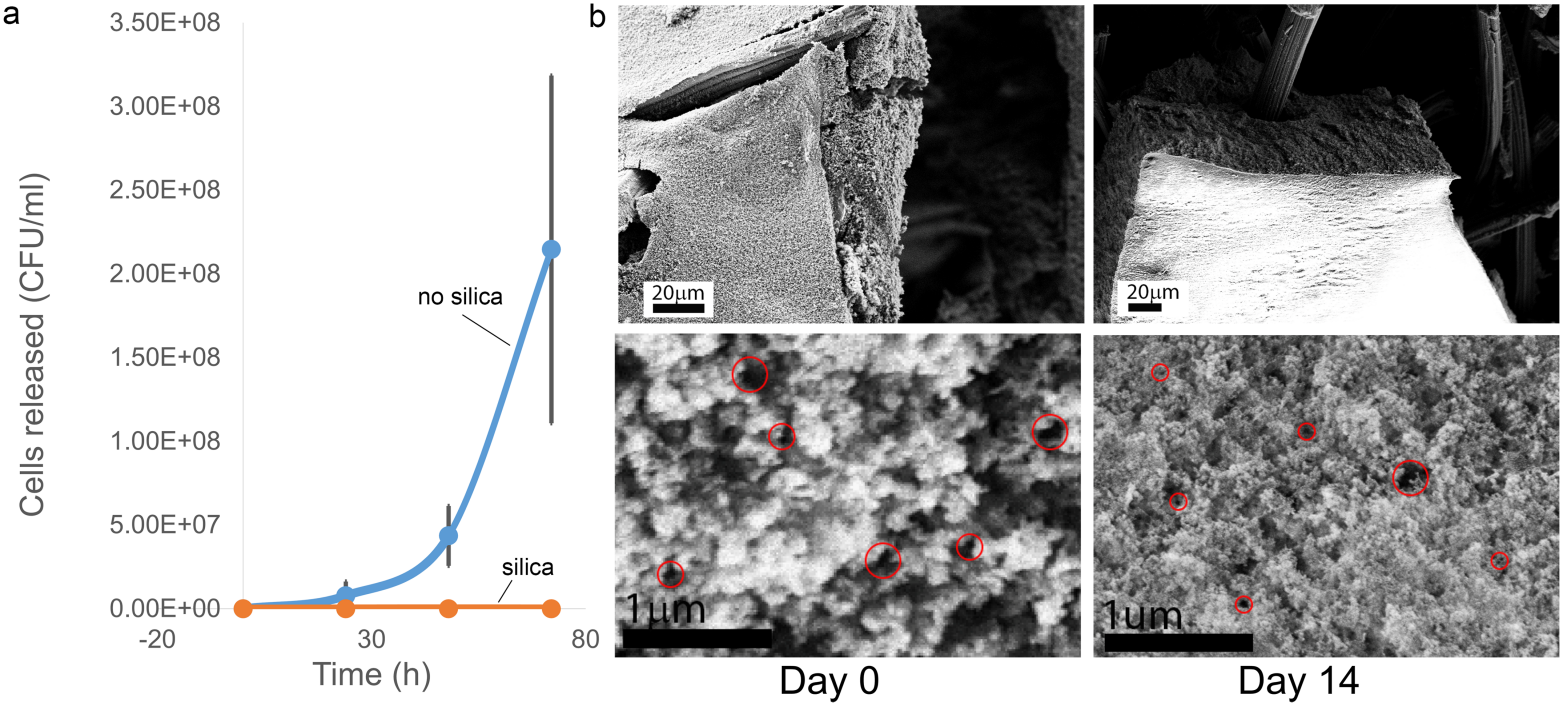
Silica filter characterization. (a) The effectiveness of silica in containing bacteria is tested by CFU tests with samples taken at t = 0, 24, 48, and 72 hours. The bare electrode (blue) shows leakage of bacteria through the electrode whereas the silica on the coated electrode (red) acts as a filter, preventing this contamination. (b) Scanning electron micrographs (SEM) of electrode surface showing the silica coating (1μm thick, 2 hour deposition) and further magnification to determine that there is evidence for pores with <0.2μm (samples circled in red), and the absence of >2μm pores taken at t = 0 and t = 14 days. Absence of significant visual discrepancies indicates the coating does not degrade over the lifetime of these experiments.

To probe the ability of the silica layer to act as a size-selective filter, we scored the extent to which the bacteria diffuse out from the m-reactor. We performed colony forming unit (CFU) tests on the surrounding medium for both silica-coated (n = 3) and naked, agarose only (n = 3), m-reactors over a 72 hour time period. Without the silica coating, the number of CFUs rapidly increase, indicating the bacteria diffuse out through the agarose and contaminate the environment. In contrast, the electrodes coated with silica did not show significant change in the number of CFUs past t = 0 h (Fig 4a). This demonstrates silica’s effectiveness in containing the bacteria inside the m-reactor and preventing contamination of the surrounding medium by our genetically modified bacteria. It also suggests that the assembly will prevent bacteria of similar or larger sizes in the environment from infiltrating our device. We acknowledge that bacteria of smaller sizes may infiltrate our m-reactor, and further research should be done on these interactions for application environments with native microbes.

To ensure that this silica filter does not erode over the course of the experiment, we took scanning electron micrographs (SEMs) of our electrode immediately after silica deposition and again after two weeks of experiments (Fig 4b). These images show robust silica encapsulation with evidence for <200 nm pores and the absence of >2 μm pores. Moreover, the multiple layers of the porous silica surface form tortuous paths, confirming that the silica acts as a filter confining the bacteria, typically 2–3 μm in length and 0.4–0.7μm in diameter [33]. The consistency of the silica coating at these time points also demonstrates the filter does not visibly disintegrate over the course of the experiment and maintains containment. The current silica deposition process results in a ~1μm thick silica layer. This is adequate for the containment of bacteria for the lifetimes of experiments in this application, but it should be noted that altering evaporation time, temperature, and pressure conditions controls silica thickness and conformality, which can be engineered for containment of bacteria of different sizes. Thus, these m-reactors offer a versatile platform for deployment into almost any aquatic environment for analyte sensing.

### Miniaturized BESSY can independently monitor two m-reactors

**Fig 5:**
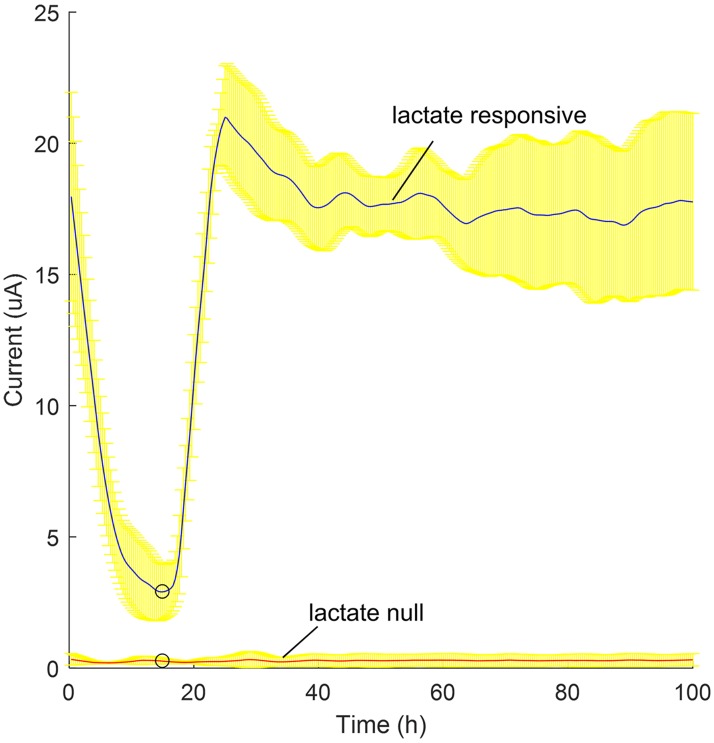
Lactate sensing platform. Average lactate responsive (blue) and lactate null (red) current response to 40mM lactate injection at t = 16h (**o**), with standard deviations (yellow) for three technical repeats (repeated at the same time) of one of two similar biological repeats (bacteria grown on different days). The ~19 μA increase in current demonstrates the microbe’s ability to reduce the carbon fibers while allowing environmental chemicals to diffuse in and out of the silica coated electrode. It also demonstrates sensing of multiple channels using one device without crosstalk.

To validate our system's ability to accurately monitor electrochemical signals from separate m-reactors without cross-talk, we measured the lactate response signals for lactate responsive (wild-type) and lactate null (*ΔmtrB*) strains of *S*. *oneidensis*. As expected [26], reactors populated with the lactate null strain produced low current and did not respond to lactate addition. In contrast, the lactate responsive assembly initially showed a decrease in current from nutrient starvation in minimal M9 medium and then responded to 40mM lactate injection with a ~19 μA jump in current (Fig 5). This demonstrates the BESSY’s ability to perform three-electrode electrochemical amperometry with our novel m-reactor design. To confirm portability, the m-reactors were overnight-air shipped with frozen packs 4,500 km to collaborators. They were able to replicate our experiment and demonstrate a current response significantly above signal noise, indicating the reactors can be stored and shipped to remote sites. These data also indicate that (1) the electrogenic bacteria are able to reduce the carbon fibers which remain in conductive contact with the titanium working electrode, (2) small chemicals, in this case lactate, can diffuse into the assembly, and (3) the system can independently record two channels using the switching mechanism. This meets the additional three requirements necessary for a sensing platform.

### BESSY can detect a chemical signal amongst environmental perturbations

**Fig 6:**
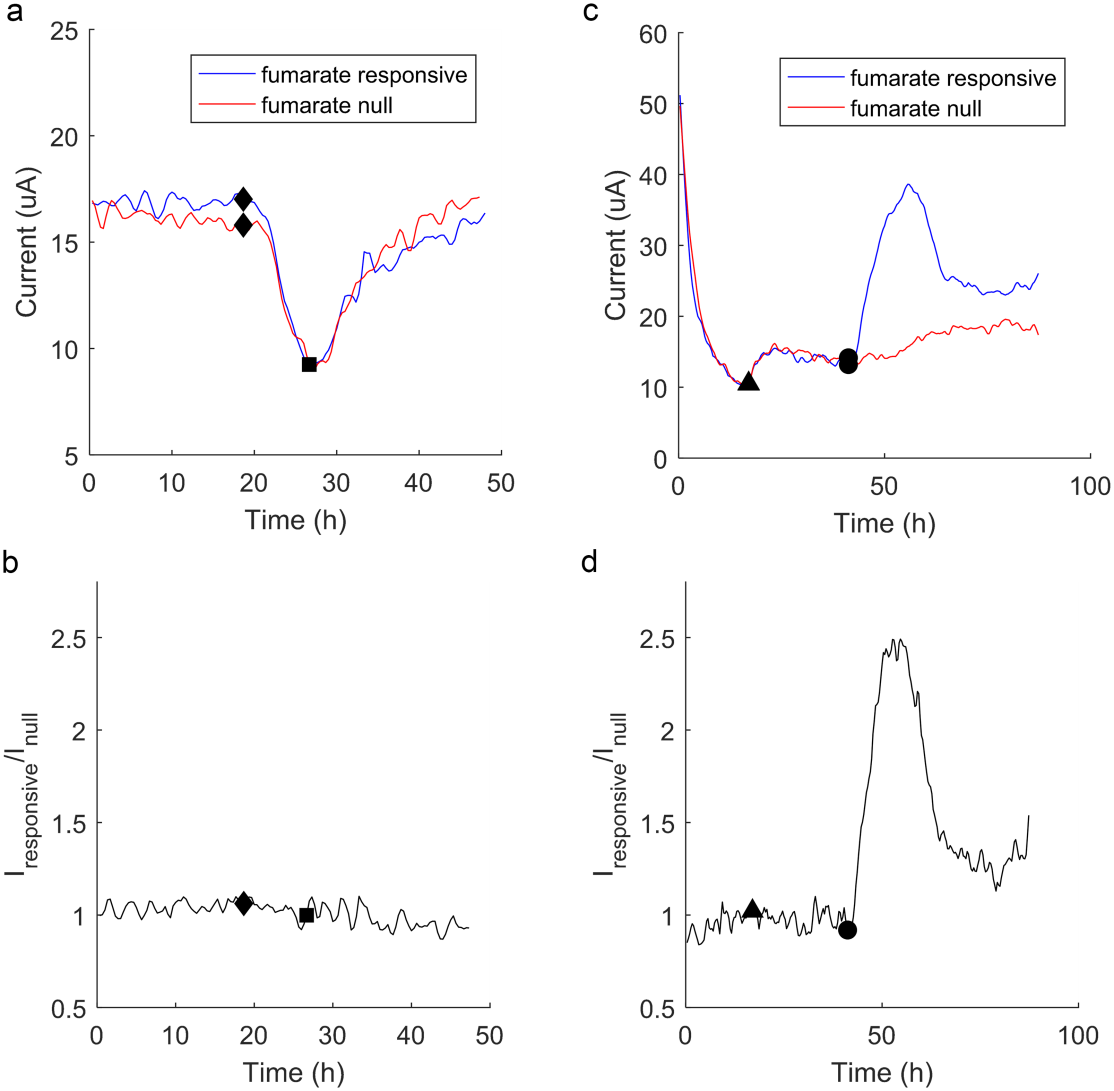
Fumarate BESSY responds amongst chemical and temperature perturbations. (a) Current in response to temperature fluctuations over time, simulated by immersing in an ice bath (◆) and removal from ice bath (**■**), shows both strains decrease and increase current production similarly. (b) The ratio of the two strains as a function of time does not change significantly, with RMS noise ~0.05. In this way, the sensor can filter out environmental variables such as temperature when deployed for environmental sensing. (c) Current in response to chemical perturbations over time generates no significant response when 10 mM lactate is added (**▲**), and a significant differential response when 1mM fumarate is added (⚫). (d) The ratio of the two currents shown above as a function of time. As expected, there is no significant fluctuation in the ratio upon addition of lactate, which contrasts the fumarate response. Figures shown here are representative of two biological repeats, each with three technical repeats (S2 Fig). Response time, defined by time to reach two standard deviations above the average environmental noise, is 1.8±0.7 hours, and the maximum ratio of I_responsive_/I_null_ is 2.1±0.4.

Our system is designed to monitor electrochemical signals from two bacterial strains with different sensing abilities to filter out chemically-induced responses from environmental variables such as temperature. To determine the BESSY’s ability to ignore environmental changes, we created a fumarate sensor employing fumarate null (*ΔfccA*) and fumarate responsive (wild type) strains of *S*. *oneidensis* and observed the effects of temperature on this sensor. The fumarate sensor was cooled to 4°C with an ice bath for 8 hours and warmed back to room temperature to imitate extreme fluctuations that the sensor could experience in deployment (Fig 6a). Both strains of *S*. *oneidensis* showed decreased and increased current production with the fall and rise of temperature, respectively. Indeed, calculating the ratio of the current produced by the analyte responsive strain over the null strain (I_responsive_/I_null_) shows that this ratio did not significantly change upon cooling or heating (Fig 6b). These controlled experiments indicate environmental fluctuations result in RMS noise of approximately 0.05 I_responsive_/I_null_. Any target analyte will have to instigate a response greater than this noise floor in order to be detected.

To test the BESSY’s ability to selectively sense one target analyte amongst other chemicals, we probed the ability of our fumarate sensor to distinguish between fumarate and lactate. When lactate is introduced at t = 20 h, both strains in the BESSY produce more current (Fig 6c) and the ratio between the currents does not change significantly (Fig 6d), which confirms the BESSY’s ability to ignore chemicals of disinterest. The RMS noise of the ratio up until the injection of fumarate continues to be approximately 0.05 I_responsive_/I_null_.

Because fumarate is an alternative electron acceptor to the metal electrode [34,35], we expect to see a decrease in current from the wild type (fumarate responsive) strain since the fccA reduces the fumarate directly. Moreover, because fumarate is consumed in this process, we expect only a temporary current response. On the contrary, little response is expected in the *ΔfccA* (fumarate null) strain due to its inability to reduce fumarate [36]. As expected, only the fumarate responsive strain changes its current production upon the addition of fumarate. When 1 mM fumarate is injected at t = 45 h, there is a significant increase in the ratio which decreases, presumably once the fumarate has been consumed, around t = 65 h. The response time of this system to fumarate, defined by time to reach two standard deviations above the average environmental noise, is 1.8±0.7 hours. At this time, we can be 95% confident that the BESSY has detected fumarate, and the signal is not fluctuating due to a change in temperature or lactate concentration. The maximum ratio of I_responsive_/I_null_ is 2.1±0.4 following the injection of fumarate. Through these experiments, we have proof of concept that differential sensing is a valid method for normalizing microbial biosensing to uncontrollable environmental variables. Moreover, we have demonstrated the functionality of this methodology on a miniaturized platform for deployable sensing.

However, it is surprising that the wild type *S*. *oneidensis* always showed a positive change in current upon addition of fumarate in our system. We speculate that this is a result of the switching feature imposed on both channels: because only one working electrode is poised at a favorable potential at a time, the bacteria in the channel which is “off” are without an electron acceptor for ten minutes at a time. Therefore, when the alternate electron acceptor, fumarate, appears in the solution, the fumarate-responsive bacteria can metabolize continuously. We suspect these conditions result in higher overall current production in the fumarate-responsive strain. This reasoning suggests that utilization of some sensing strains within our system may yield in changes in I_responsive_/I_null_ that have the opposite sign as what is observed when performing continuous monitoring. It is important to note that for the application of detection of target chemicals between an analyte sensing and null strain, as is demonstrated here, the direction of change is not as important as a proportional change in current. By setting a threshold for the ratio of I_responsive_/I_null_, in this case ~1.5, the system can alert when the ratio peaks. Depending on the application, this threshold can be set to be very conservative, alerting the user at the slight possibility of signal, or rather liberal in monitoring uses where the chemical is not hazardous. Using the BESSY with an engineered selection of analyte sensitive electrogenic bacteria, we can make our device specific to only our target analyte and detect fumarate amongst environmental perturbations, such as temperature and variations in other chemicals.

## 4. Conclusion

We have demonstrated a miniaturized alternating potentiostat capable of using differential signal measurements to detect fumarate amongst other environmental variables. We have also designed a small, portable m-reactor to contain electrogenic bacteria without contaminating its surroundings. This 2x1x1 cm assembly is more than 10x smaller in volume than other submersible microbial biosensors [37,38]. Moreover, most of the currently existing *in situ*, biosensors focus on portability of the reactor assembly but still rely on external electronics, such as a commercial multimeter [39,40], and also require calibration for different environmental conditions such as pH and temperature [37]. By miniaturizing the electronics and incorporating an analyte sensing and null strain, this work introduces the concept of a fully mobile, easily deployable BESSY (bioelectronic sensing system) for environmental sensing. The differential sensing system enables self-calibration in various environments, in the same way that we show for temperature and variability in nutrients (lactate), provided the same type of differential correction could be made for changes in pH, alkalinity, and other conditions. This biosensing platform is not without challenges, though, which include hour-scale response times for transcriptional regulation based sensors, long times to return to baseline, and the requirement for adequate growth nutrients in the environment [8,9]. These drawbacks will limit the deployment of whole-cell electrochemical biosensing technology, but improvements can also be made by focusing on allosteric response pathways [41] or integrating nutrient reservoirs into the device [42].

Applications for this biosensing platform extend far beyond fumarate and are only limited by the chemicals we can genetically engineer electrogenic bacteria to be sensitive to. With the development of arsenic [19] and arabinose [20] sensing *S*. *oneidensis*, as well as introduction of the exoelectrogenic MR-1 Mtr pathway to *E*. *coli* [43–46], there are numerous opportunities to sense a variety of vital chemicals by engineering the outer membrane protein complexes. Selectivity and sensitivity of the BESSY will be greatly dependent on which of these sensing strains is chosen to be incorporated into the system. As shown with the fumarate sensing system, it is possible to sense analytes in the presence of active metabolism. Further work could be pursued through additional miniaturization and reduction of power of (1) the abiotic core through ASIC design, and (2) the biotic component by customization of reference electrode and using a smaller volume reactor. The ability to interface low power electronics with robust, sensing, exoelectrogenic bacteria such as *S*. *oneidensis* shows potential in efforts towards continuous, real-time monitoring of aquatic environments.

## Supporting information

S1 Fig Comparison of our COTS potentiostat with a commercial potentiostat (BioLogic VSP-300). The COTS potentiostat is accurate to within 10% error from the commercial potentiostat over a range of 10uA to 1.5mA. A Kiethley 2400 Sourcemeter was used to sweep a known current through the device in a two-electrode configuration.
(TIF)

S2 Fig Temperature and lactate response for three technical repeats. (a) Current in response to temperature fluctuations over time, simulated by immersing in an ice bath (o) and removal from ice bath (+), shows a similar effect on both strains of bacteria, resulting in an RMS noise of approximately 0.05 Iresponsive/Inull. (b) Current in response to chemical perturbations over time illustrates an insignificant response when 10 mM lactate is added (o), and a significant differential response when 1mM fumarate is added (+). The ratio shows an average response time of 1.8±0.7 hours, and the maximum ratio of Iresponsive/Inull is 2.1±0.4.
(TIF)
