# Supplementary figures and images for: A portable bioelectronic sensing system (BESSY) for environmental deployment incorporating differential microbial sensing in miniaturized reactors
Source: PLoS One. 2017 Sep 15;12(9):e0184994. doi: 10.1371/journal.pone.0184994 (PMC5600388; doi:10.1371/journal.pone.0184994)

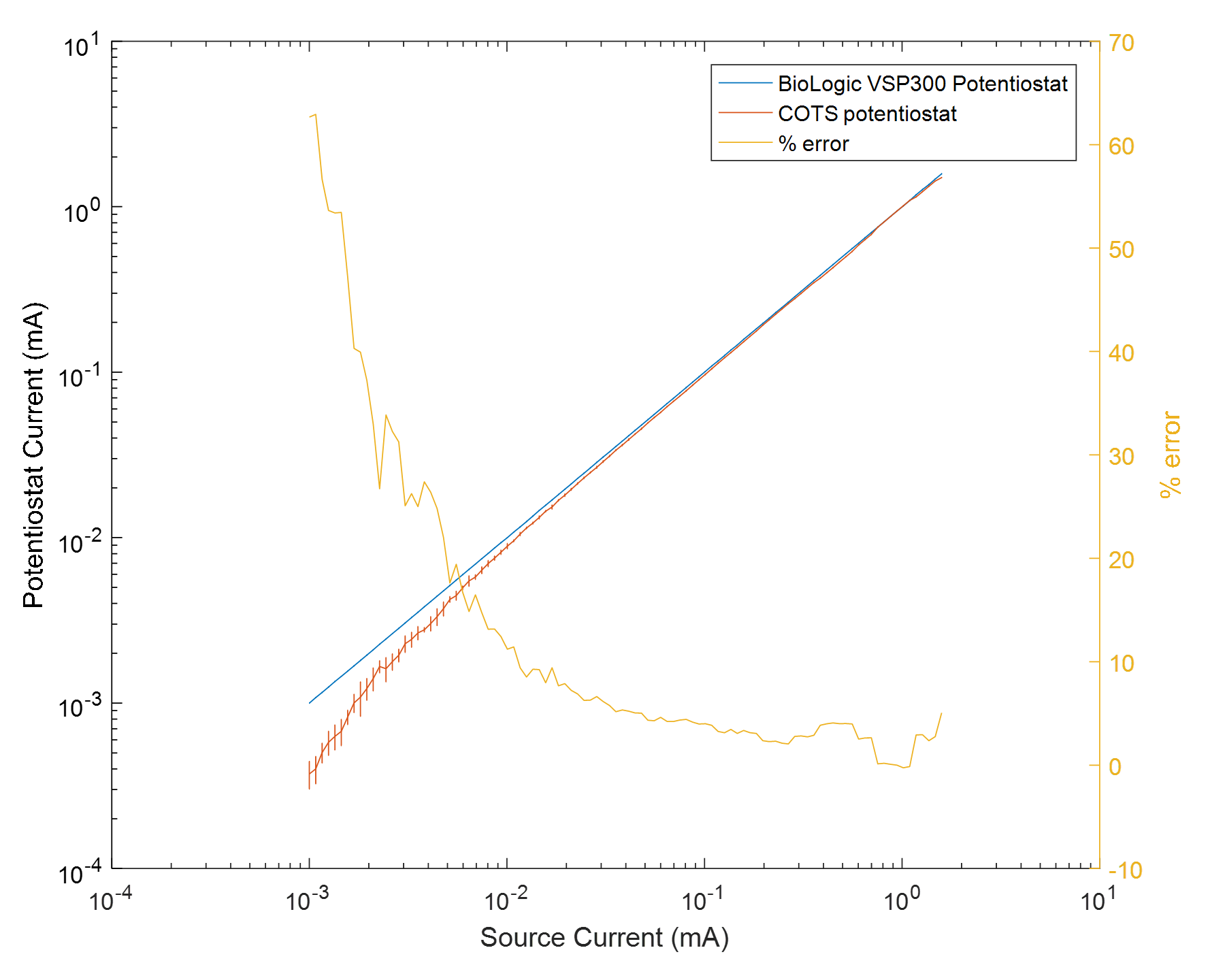

Supplement: S1 Fig — The COTS potentiostat is accurate to within 10% error from the commercial potentiostat over a range of 10uA to 1.5mA. A Kiethley 2400 Sourcemeter was used to sweep a known current through the device in a two-electrode configuration. (TIF) [file pone.0184994.s001.tif]

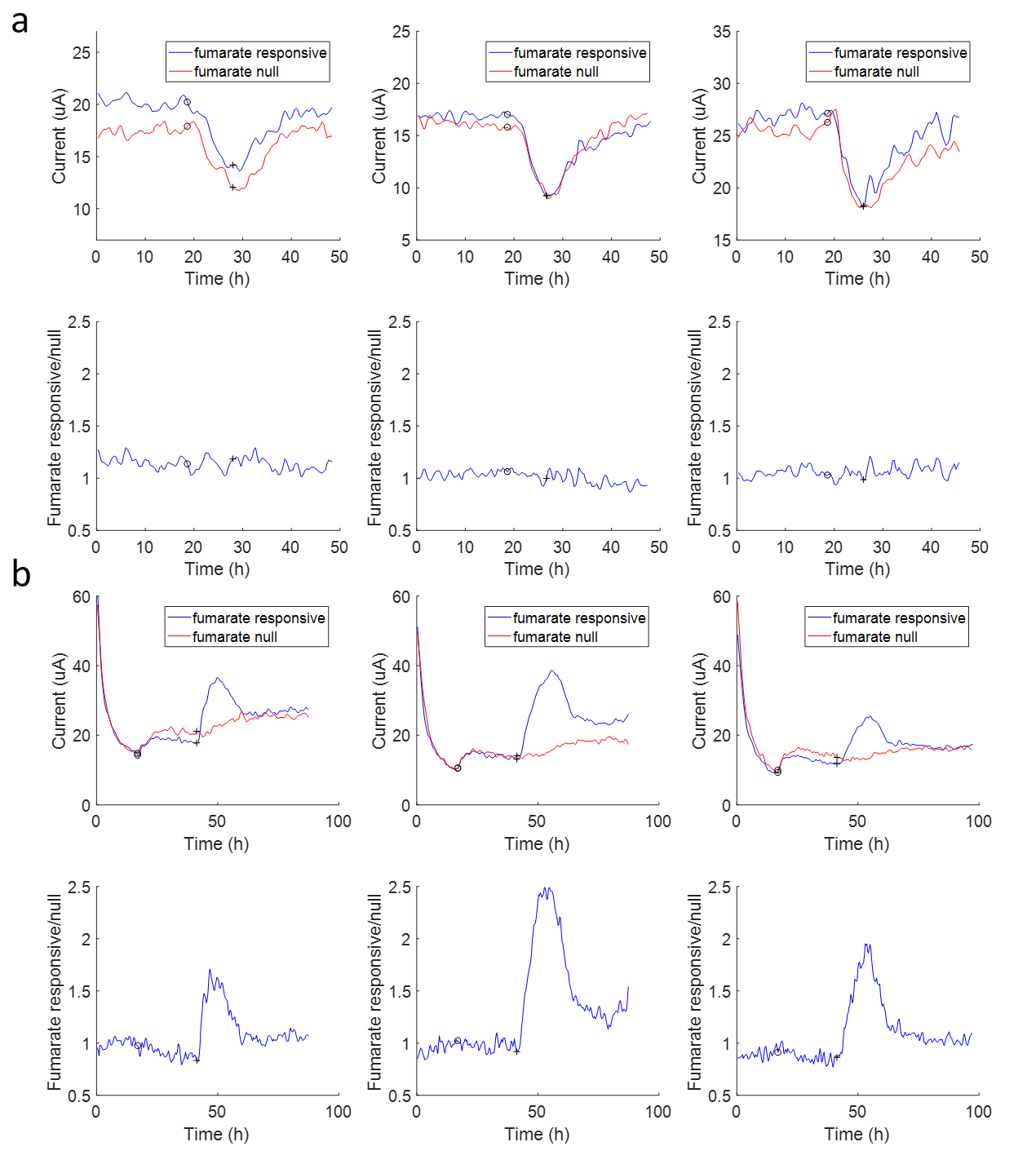

Supplement: S2 Fig — (a) Current in response to temperature fluctuations over time, simulated by immersing in an ice bath (o) and removal from ice bath (+), shows a similar effect on both strains of bacteria, resulting in an RMS noise of approximately 0.05 Iresponsive/Inull. (b) Current in response to chemical perturbations over time illustrates an insignificant response when 10 mM lactate is added (o), and a significant differential response when 1mM fumarate is added (+). The ratio shows an average response time of 1.8±0.7 hours, and the maximum ratio of Iresponsive/Inull is 2.1±0.4. (TIF) [file pone.0184994.s002.tif]
